# Prospective association between perceived stress and anxiety among nursing college students: the moderating roles of career adaptability and professional commitment

**DOI:** 10.1186/s12888-023-04887-6

**Published:** 2023-06-01

**Authors:** Chaoqun Dong, Lin Xia, Chengjia Zhao, Xiaoxian Zhang, Ju He, Guohua Zhang, Jingjing Zhang

**Affiliations:** 1 School of Nursing, Wenzhou Medical University, Wenzhou, 325035 China; 2 School of Education, Renmin University of China, Beijing, 100872 China; 3 Department of Psychology, School of Mental Health, Wenzhou Medical University, Wenzhou, 325035 China; 4 The Affiliated Kangning Hospital, Wenzhou Medical University, Wenzhou, 325035 China

**Keywords:** Nursing college students, Perceived stress, Anxiety, Career adaptability, Professional commitment

## Abstract

**Background:**

Anxiety may stay with nursing students throughout their internship and even persist afterwards. Although many studies have explored the effects of perceived stress on anxiety, the relationship between pre-internship perceived stress and post-internship anxiety levels has not been clarified. In addition, none had focused on the moderating roles of career adaptability and professional commitment between perceived stress and anxiety. This study aims to investigate the influence of pre-internship perceived stress on the post-internship anxiety level of nursing college students, and to analyze the moderating effects of career adaptability and professional commitment on their relationships.

**Methods:**

A longitudinal study design was employed. Full-time nursing college students from a Chinese medical university were recruited by convenient sampling. All surveys were conducted via Wen Juan Xing (www.wjx.cn), a widely used web-based survey platform in China. Two waves of surveys were collected in the pre-internship and post-internship periods, with an interval of one year. Among 823 nursing students recruited, 692 students completed all two waves of the survey (response rate: 84.08%). Participants completed a series of questionnaires examining general demographic characteristics, perceived stress, anxiety, career adaptability, and professional commitment both before and after the internship. The bias-corrected bootstrap technique of the Hayes PROCESS macro (Model 2) was used to test the moderation effect.

**Results:**

Pre-internship perceived stress was positively associated with post-internship anxiety (*β* = 0.474, *p* < 0.001). Career adaptability would mitigate the effect of perceived stress on anxiety (*β* = -0.009, *p* < 0.01, 95% CI = [-0.013, -0.004]), and this influence became stronger for nursing college students with higher levels of career adaptability. Instead, the professional commitment would enhance the effect of perceived stress on anxiety (*β* = 0.004, *p* < 0.05, 95% CI = [0.001, 0.009]).

**Conclusions:**

Adequate career adaptability was key to alleviating anxiety among nursing interns. Nursing educators and clinical nursing managers should pay attention to cultivating the career adaptability of nursing college students in order to help them successfully achieve identity transformation and career development. Meanwhile, it is crucial to guide them to develop appropriate professional commitment.

## Introduction

As the driving force of the modern healthcare system, nurses perform a vital role in promoting public physical and mental health. However, due to a variety of circumstances including doctor-patient conflicts, intense workload, and prevention and management of infectious diseases, nurses generally experience high levels of psychological stress and are prone to job burnout or even resignation [1–3]. This issue has been compounded by the COVID-19 pandemic [4]. The massive loss of nursing talents will result in an inadequate supply of nursing services and exacerbate the medical and health resource shortage in China. Nursing college students are the reserve force of future hospital nurses, thus nursing education and training to enhance students’ skills will be beneficial for addressing the nurse shortage issue [5].

Clinical practice in hospitals is an essential part of the undergraduate nursing program to prepare students to be well-equipped for their future careers. In China, nearly all nursing students go to hospitals for at least 10-month, full-time clinical practice in their final years [6]. Hence, clinical practice can be viewed as a stage of career preparation and identity transition from student nurses to registered nurses. Nonetheless, nursing students may encounter numerous unpredictable challenges during their clinical practice, such as a lack of clinical experience [7], the complexity of interpersonal relationships [8], and the inability to adapt to the new learning environment [9], which will cause significant psychological stress and may result in anxiety [10]. According to previous studies, all nursing students who participated in clinical practice experienced a considerable level of stress and anxiety, and their anxiety levels were significantly greater than those of other college students [11, 12]. Anxiety is usually present throughout the entirety of the clinical practice process, and the level of anxiety fluctuates throughout the stages of practice [13]. High levels of anxiety can interfere with the learning efficacy in clinical practice, impair academic achievement, and result in serious mental health problems [9, 14]. If nursing students continue to experience excessive anxiety after clinical practice without developing the proper coping ability, this will negatively affect their willingness to become nurses, career planning, and decision-making [13, 15]. Factors influencing the anxiety level of nursing college students are complex and varied, including demographic characteristics (such as gender, age, hometown location, whether a one-child family or not, and parents’ educational level) [16–18], perceived stress [19], career adaptability [20], professional commitment [21]. Although several studies have explored the relationship between the above-mentioned factors and anxiety, little is known about the mechanisms of how perceived stress, career adaptability, and professional commitment contribute to nursing students’ anxiety during clinical practice.

Many previous studies have confirmed that stressful events are important predictors of negative emotions such as anxiety and depression [22, 23]. Full-time nursing college students lack clinical practice experience during their college years, so nursing students may encounter a variety of stressors before their internship, such as concern about their lack of professional knowledge and skills, lack of confidence to be competent in clinical work, worry about the complexity of interpersonal relationships, and fear of making mistakes and being judged [12, 24, 25]. Individuals’ perception of stress plays an important predictive role in the transmission and induction of anxiety by stressors [26, 27]. Perceived stress is an individual’s subjective response to various stimuli and threats in the environment after their cognitive evaluation [28, 29]. Individuals who have experienced stressful events will evaluate the severity of the crisis perceptually and cognitively, resulting in a certain degree of physiological and psychological reactions [30]. The appraisal theory of emotion suggests that specific emotional reactions arise from the individual subjective appraisal of the stressor rather than the stressful events perse [31]. Individuals who perceive stress as a threat may become unproductive, resulting in negative emotions such as anxiety [30]. Several empirical research has also demonstrated a correlation between higher levels of perceived stress and higher levels of anxiety [32, 33, 30]. However, due to the cross-sectional design of previous studies, whether the perceived stress before clinical practice can predict post-internship anxiety remains unknown.

The transactional theory of stress and coping [34] suggests that individual evaluation of situation and self-ability plays a key role in the process of stress coping, and the interaction between individual and environment affects the behavior and response of stress. Hence, career adaptability and professional commitment as the individual’s psychosocial construct [35] or attitude that provides a physical, mental, and emotional connection to work and professional behaviors [36] may be the potential mechanism that affects the relationship between perceived stress and anxiety.

Career adaptability refers to individuals’ ability to adapt and cope with unpredictable events in their careers, and to maintain the balance of career roles [37]. Moreover, it is an essential ability for nursing students to grow up into excellent nursing professionals [38]. According to the Career Construction Model of Adaptation, career adaptability can influence career-related behaviors with positive and negative emotions as the important outcomes of adaptive behaviors [39]. Individuals who have difficulty adapting to anticipated and unanticipated career-related challenges may experience anxiety and other negative mental health outcomes [40]. A previous study found that career adaptability had an impact on undergraduate students’ future career decisions and anxiety levels [41]. Individuals with higher career adaptability are better able to manage negative emotions when in career-related environments or dealing with career-related matters [40], for those with high career adaptability usually have a stronger desire for their ideal career, a higher willingness to make efforts, and a greater sense of control over various unpredictable situations in the career [42]. When nursing students perceive greater stress, adequate adaptation and coping capacity may enhance their learning motivation and confidence in problem-solving, consequently reducing their anxiety levels [43]. Thus, career adaptability may moderate the effect of perceived stress on the anxiety of nursing students.

Professional commitment is defined as “individuals’ attitude towards and identification with their profession or career” or “individuals’ motivation for career choice and loyalty to career” [44]. Individuals with high professional commitment are usually characterized by a propensity to identify with their profession, a strong belief in the goals and values of their profession, and an increased willingness to pursue a professionally relevant career [44, 45]. The professional commitment was believed to moderate the negative effects of a high-pressure workplace [46], and studies have shown that nurses who had a strong sense of professional identity and professionalism were less likely to feel stressed at work [21, 47]. It has been proposed that professional commitment may be a protective factor against adverse psychological reactions [48]. Stress is experienced when clinical practice settings are judged to be beyond one’s capabilities [45]. Individuals with a higher level of professional commitment will be more motivated to use various resources to cope with difficulties, which helps them to face the clinical practice environment with a more optimistic attitude, thus reducing adverse psychological reactions [49]. Previous research has also found a close association between increased professional commitment and lower levels of anxiety [21]. However, there is a lack of empirical studies to identify whether professional commitment can moderate the relationship between perceived stress and anxiety.

**Fig. 1:**
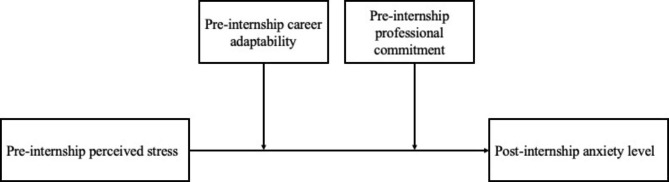
The proposed conceptual model

Therefore, this study aimed to investigate the influence of pre-internship perceived stress on the post-internship anxiety level using a longitudinal design and to analyze the moderating effects of career adaptability and professional commitment on the relationship between pre-internship perceived stress and the post-internship anxiety level among nursing college students, which may provide beneficial guidance for targeted intervention to alleviate the anxiety of nursing students in the clinical practice. The present study hypothesized that (see Fig. 1): (1) pre-internship perceived stress was positively correlated to post-internship anxiety level (H1); (2) career adaptability moderated the relationship between pre-internship perceived stress and post-internship anxiety level (H2); (3) professional commitment moderated the relationship between pre-internship perceived stress and post-internship anxiety level (H3).

## Methods

### Sample and research design

Full-time college students from a Chinese medical university were recruited by convenient sampling. Participants who met the inclusion criterion were invited to participate in this study. College students were eligible if they (1) majored in nursing, (2) had completed all major courses before clinical internship , (3) started the internship during the summer of 2021 and ended their internship during the spring of 2022, and (4) volunteered to participate in this study. Participants who have less than 10 months of internship would be excluded. Two waves of surveys were collected in the pre-internship and post-internship periods (June 2021/June 2022), with an interval of one year. Among 823 nursing students recruited, 795 students completed the first wave survey and 692 students completed all two waves of the survey (response rate: 84.08%), respectively. The sample size should be at least 10 times more than the number of paths to be estimated in the model [50]. Participants have exceeded the required sample size for this model (3*10 = 60). All surveys were conducted via Wen Juan Xing (www.wjx.cn), a widely used web-based survey platform in China. A well-trained and experienced research assistant explained to participants that their participation was voluntary, and refusal to participate would not result in any negative consequences. The data confidentiality was guaranteed and only the researchers have access to the data. Student IDs were collected for data matching. Researchers were not able to access students’ names or other identifying information.

### Measures

#### General demographic questionnaire

The general demographic questionnaire collected information including gender, age, hometown location, whether a one-child family or not, and parents’ educational level.

#### Perceived stress scale

The 14-item Perceived Stress Scale [28] was used to assess participants’ level of pre-internship perceived stress. Item responses are scored on a 5-point Likert scale (0 = never and 4 = very often) with seven items for reverse scoring. The total score is a sum of 14 items (ranging from 0 to 56), with higher scores meaning more perceived stress. The scale had good reliability and validity in the Chinese population [51, 52]. In the present study, Cronbach’s α was 0.79.

#### Career adaptability scale

The Career Adaptability Scale developed was used to measure the pre-internship career adaptability of nursing students [53]. It is composed of 21 items with four dimensions: career confidence, career curiosity, career concern, and career control. Items are rated on a 5-point Likert scale from 1 (strongly disagree) to 5 (strongly agree). The total score ranges from 21 to 105, with higher scores indicating greater career adaptability. The psychometric properties of this scale have been widely confirmed in Chinese college students [54, 55]. In the present study, Cronbach’s α was 0.95.

#### Undergraduates’ professional commitment scale

The Undergraduates’ Professional Commitment Scale [56] was used to assess the pre-internship professional commitment of nursing students. This 27-item scale includes four dimensions: affective commitment, continuance commitment, ideal commitment, and normative commitment. Item responses are scored on a 5-point Likert scale (1 = strongly disagree, 5 = strongly agree) with higher scores indicating greater levels of professional commitment. This scale had good reliability in the previous study [57]. In the present study, Cronbach’s α was 0.96.

#### Hospital anxiety and depression scale

The Hospital Anxiety and Depression Scale developed by Zigmond and Snaith [58] was translated into the Chinese version by Ye et al. [59], which consists of an Anxiety subscale (HADS-A) and a Depression subscale (HADS-D). The HADS was widely used in general populations [60, 61] such as students [62], elderly people [63], and community residents [64], and has been proven to be a valid instrument for measuring anxiety and depression, with good reliability and validity. The HADS-A was used to assess participants’ post-internship anxiety levels in this study. The HADS-A contains 7 items with each item scoring on a 4-point Likert scale from 0 to 3. The total score of HADS-A ranges between 0 and 21 points, with higher scores indicating more severe anxiety. The following cutoff points were employed: a score of 0–7 means no anxiety, 8–10 indicates mild anxiety, 11–14 shows moderate anxiety and 15–21 indicates severe anxiety. The good reliability of HADS-A has been confirmed in previous studies [65, 66]. In the current study, Cronbach’s α was 0.88.

### Statistical analyses

Statistical analyses were conducted with the Statistical Package for the Social Sciences 23 (SPSS, New York, NY, USA) and the PROCESS macro version 3.3 (www.Processmacro.org/index.html) which is the Process macro from SPSS. All variables of interest were normally distributed; the skewness and kurtosis of pre-internship perceived stress, pre-internship career adaptability, pre-internship professional commitment, and post-internship anxiety fell within the acceptable range [i.e., skewness < |2.0| and kurtosis < |7.0|]. All variables were standardized before analyses. Multicollinearity was not a problem according to the variance inflation factors. Descriptive data are presented as Mean (standard deviation, SD) and range. Correlation analysis was performed between relevant variables. Our research adopts bias-corrected bootstrapping techniques by Hayes PROCESS macro (Model 2) to test the moderation effect. The parameter was significant if the 95% bias-corrected confidence interval (CI) did not contain zero after 5000 bootstrapped samplings.

### Ethical approval and informed consent

Informed consent was obtained from all participants for this study. This study received approval from the Ethics Committee of Wenzhou Medical University (code: 2022-028).

## Results

### Descriptive analyses and bivariate analyses

A common method for bias testing was performed. According to Harman’s single-factor test, 50 principal components were extracted without rotation, and the explanatory rate of the total variance variation of the first was 37.4%, lower than the critical value of 40.0% [67]. Thus, the data had no severe common bias.

Of these 692 participants who completed the two-wave survey, 618 (89.3%) were female, 181 (26.2%) were from urban areas, and 227 (32.8%) were from one-child families. The mean age of the participants was 20.94 years (SD = 1.79, range = 19–24 years). In terms of parents’ educational level, the majority of fathers (n = 485, 70.1%) and mothers (n = 538, 77.7%) had junior middle school or lower level, and 207 (29.9%) and 154 (22.3%) of their fathers and mothers were with senior middle school level or above, respectively.

Table 1 shows the means, SDs, and correlations for all variables. As the results showed, pre-internship perceived stress was positively correlated with post-internship anxiety (*r* = 0.43, *p* < 0.001), pre-internship perceived stress was negatively correlated with pre-internship career adaptability (*r* = -0.69, *p* < 0.001) and pre-internship professional commitment (*r* = -0.55, *p* < 0.001). Post-internship anxiety was negatively correlated with pre-internship career adaptability (*r* = -0.28, *p* < 0.001) and pre-internship professional commitment (*r* = -0.27, *p* < 0.001). The association between pre-internship career adaptability and pre-internship professional commitment was positive (*r* = 0.74, *p* < 0.001). Gender was positively correlated with pre-internship career adaptability (*r* = 0.08, *p* < 0.05), and father’s educational level was negatively correlated with pre-internship perceived stress (*r* = -0.09, *p* < 0.05), but positively correlated with post-internship anxiety (*r* = 0.09, *p* < 0.05). Mothers’ educational level was negatively correlated with pre-internship perceived stress (*r* = -0.11, *p* < 0.01), but positively correlated with post-internship anxiety (*r* = 0.12, *p* < 0.01) and pre-internship career adaptability (*r* = 0.08, *p* < 0.05).

**Table 1:** Descriptive statistics and correlations among variables of interest (N = 692)

	M	SD	1	2	3	4
1. Pre-internship perceived stress	37.02	5.95	1	-	-	-
2. Post-internship anxiety	10.81	3.17	0.43^***^	1	-	-
3. Pre-internship career adaptability	74.33	10.48	-0.69^***^	-0.28^***^	1	-
4. Pre-internship professional commitment	94.94	13.74	-0.55^***^	-0.27^***^	0.74^***^	1
5. Gender	-	-	0.01	0.01	0.08^*^	0.07
6. Age	20.94	1.79	-0.02	-0.03	-0.03	0.01
7. Hometown location	-	-	0.07	-0.07	-0.03	-0.01
8. One-child family	-	-	-0.03	0.03	0.01	0.03
9. Father’s educational level	-	-	-0.09^*^	0.09^*^	0.05	0.03
10. Mother’s educational level	-	-	-0.11^**^	0.12^**^	0.08^*^	0.01

Note: ^*^p < 0.05, ^**^p < 0.01, ^***^p < 0.001

### Moderating effects of career adaptability and professional commitment

**Fig. 2:**
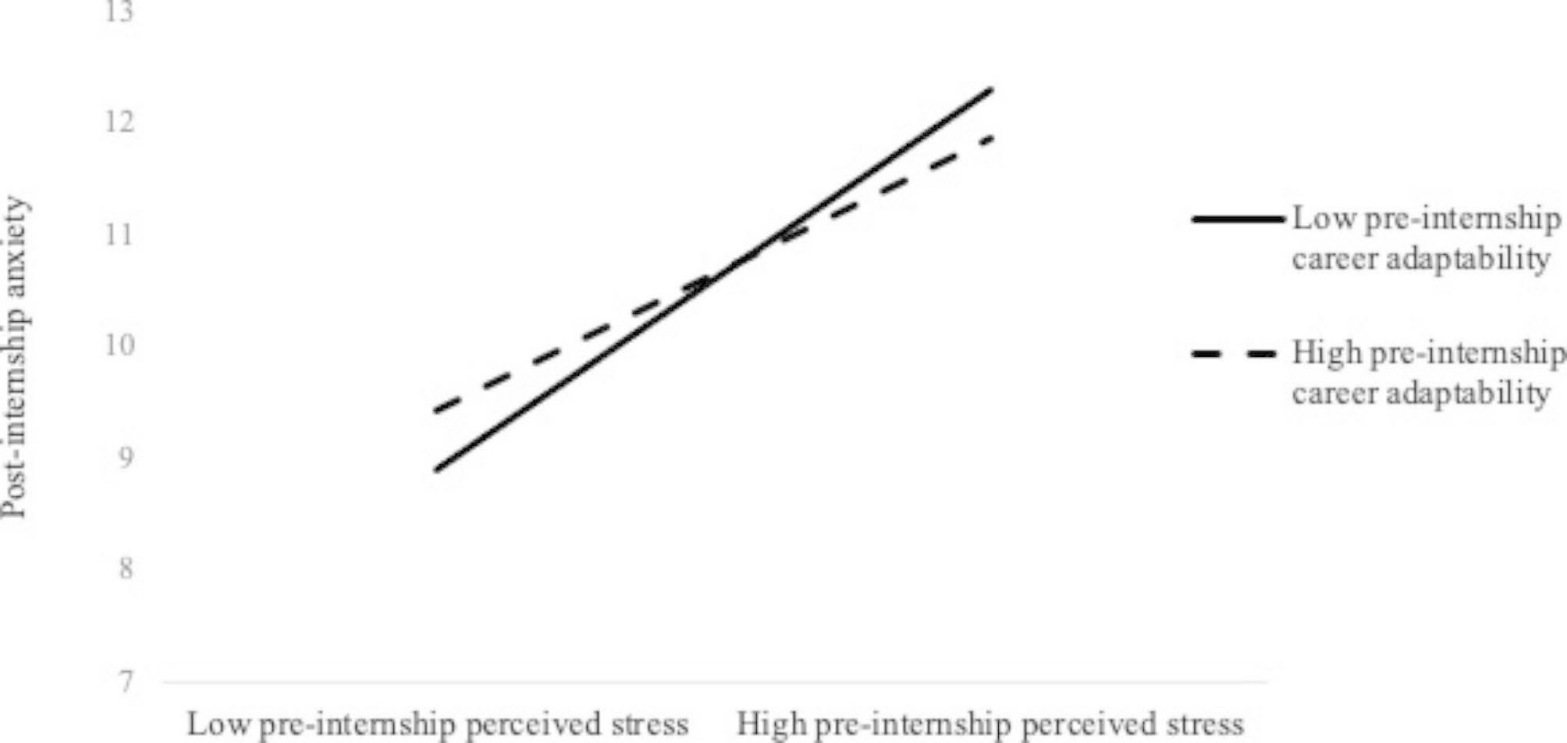
Interaction between pre-internship perceived stress and career adaptability on post-internship anxiety

Controlling for gender, father’s educational level, and mothers’ educational level, the outcomes showed that there was a significant positive correlation between pre-internship perceived stress and post-internship anxiety level (*β* = 0.474, *p* < 0.001) with 95% CI [0.252, 0.697] (Table 2). Career adaptability significantly positively predicted post-internship anxiety level (*β* = 0.330, *p* < 0.001, 95%CI = [0.167, 0.492]), and the interaction effect of pre-internship perceived stress and career adaptability can significantly predict post-internship anxiety (*β* = -0.009, *p* < 0.01, 95%CI = [-0.013, -0.004]). We plotted predicted post-internship anxiety against pre-internship perceived stress separately for low (M-SD) and high (M + SD) levels of career adaptability (Fig. 2). The positive association between pre-internship stress and post-internship anxiety was higher in nursing students with a low level of career adaptability (*β* = 0.288, t = 9.224, *p* < 0.001, 95% CI= [0.226, 0.349]), compared to those with a high level of career adaptability (*β* = 0.206, t = 7.321, *p* < 0.001, 95% CI= [0.151, 0.261]). Pre-internship professional commitment significantly negatively predicts post-internship anxiety (*β* = -0.175, *p* < 0.01, 95%CI = [-0.290, -0.060]), and the interaction effect of pre-internship perceived stress and professional commitment significantly predicted post-internship anxiety (*β* = 0.004, *p* < 0.05, 95%CI = [0.001, 0.009]). However, a simple slope test did not find significant differences.

**Table 2:** The moderating effects of career adaptability and professional commitment

	Post-internship anxiety			
Variables	*β*	SE	*t*	95%CI
Gender	0.019	0.351	0.053	[-0.670, 0.701]
Father’s educational level	0.357	0.263	1.355	[-0.160, 0.873]
Mother’s educational level	0.228	0.296	0.768	[-0.354, 0.809]
Pre-internship perceived stress	0.474^***^	0.113	4.181	[0.252, 0.697]
Pre-internship career adaptability	0.330^***^	0.083	3.983	[0.167, 0.492]
Pre-internship perceived stress * career adaptability	-0.009^**^	0.002	-3.763	[-0.013, -0.004]
Pre-internship professional commitment	-0.175^**^	0.059	-2.992	[-0.290, -0.060]
Pre-internship perceived stress * professional commitment	0.004^*^	0.002	2.701	[0.001, 0.009]
R^2^	0.211			
F	22.806^***^			

Note: ^*^p < 0.05, ^**^p < 0.01, ^***^p < 0.001

## Discussion

This study confirms the hypothesis that pre-internship perceived stress would significantly predict post-internship anxiety among nursing college students. The positive association between perceived stress and anxiety has been documented in previous studies [32, 33, 30]. According to the Trait-State Anxiety Theory [68, 69], nursing students who perceived the clinical practice as a stressful event may fall into a state of anxiety. Nursing students are still in the transitional stage of psychological and physiological development before entering the internship, and their psychological defense mechanisms are immature [13]. A previous study found individuals with lower perceived stress had a greater ability to regulate and adapt to changes in their lives and environment, thereby weakening negative experiences in work [70]. In that case, the higher perceived pressure about their future internship may make nursing students more sensitive to negative signals from the clinical environment during their internship and make it may more difficult to overcome the difficulties that arise in practical work. Therefore, it is important for nursing educators and clinical nursing managers to develop intervention strategies to reduce the pre-internship perceived pressure to improve nursing students’ positive experience and reduce their anxiety levels during clinical practice.

The results of the moderating roles of career adaptability on the relationship between pre-internship perceived stress and post-internship anxiety level supported hypothesis 2. Our results revealed that adequate career adaptability was one of the key factors to alleviate the negative effect of perceived stress on anxiety, which was in line with the previous findings that students with greater adaptability exhibited more adaptive behaviors and positive emotions amidst stress and were more capable of recovering from stress [71, 72]. These findings are consistent with Piaget’s equilibration theory [73], which emphasizes the importance of adaptation in the individual’s quest for balance. Nursing students had to face the demands of identity transformation and integration into a new professional environment at the very beginning of clinical practice [21, 74], and the high level of stress perceived in the stressful clinical environment made the equilibrium between individuals and the environment broken [9, 75]. Adequate career adaptability can help nursing interns perceive more possibilities and opportunities [76], positively cope with complex work environments and work challenges [77], improve their mental health [78], and thus better maintain career role equilibration. Our study highlights the importance of the cultivation of career adaptability in the nurse education program to help nursing students successfully adapt to the career environment.

This study revealed the moderating effects of professional commitment on pre-internship perceived stress and post-internship anxiety levels, suggesting that there was an interaction between professional commitment and perceived stress in predicting anxiety levels. Interestingly, while most current research has focused on the positive effects of professional commitment [79, 80], our study found that professional commitment may also enhance the effects of perceived stress on anxiety. There are several possible reasons for this finding. Nursing college students with higher professional commitment usually have higher expectations and requirements for personal career development and self-worth [81]. In that case, the inconsistency between the reality of stressful clinical practice, which is with the heavy workload, and high occupational pressure, risk, and intensity [82], and their idealistic career perceptions may increase their psychological burden and anxiety symptoms [6]. Moreover, a previous study [82] found that despite the high professional commitment and high desire to pursue a nursing career, nursing graduating students’ willingness to make efforts for the career is relatively low. Hence, high professional commitment cannot guarantee nursing students’ effort to manage their anxiety symptoms under perceived greater stress. The above findings suggest that nurse educators should dialectically view the influence of professional commitment and conduct educational interventions to guide nursing college students to maintain appropriate professional commitment. Unexpectedly, the simple slope test did not find significant differences between high and low levels of professional commitment in moderating the relationship between perceived stress and anxiety. This may be related to the relatively homogeneous research population due to the convenience sampling in this study. In addition, the majority of respondents in the study were female, and this gender imbalance may lead to a misrepresentation of certain associations. Future research needs to use a more diverse and heterogenous sample to further explore the role of professional commitment in the relationship between perceived stress and anxiety.

The findings of this study can be explained by the transactional theory of stress and coping [34]. The theory specifically focuses on the individual evaluation of situation and self-ability as well as the interaction between individual and environment during the process of stress coping. Nursing students with higher adaptability tend to mobilize coping resources to solve problems and adapt to environment when they perceive higher levels of stress. Meanwhile, the high-stress professional environment may exacerbate psychological burden and anxiety symptoms in nursing students who show higher commitment and loyalty to their profession. To our best knowledge, this is the first study to assess the moderating effects of career adaptability and professional commitment on the relationship between the pre-internship perceived stress and post-internship anxiety level among nursing college students. Thus, our study provides a basis for further enriching the knowledge of nursing college students , educators, and clinical administrators.

## Implications

The present study provides some beneficial implications for nursing educators and clinical nursing managers to cultivate high-quality nursing reserve talents. Firstly, our results emphasize the necessity of nursing educators preparing their students with sufficient coping skills to reduce perceived stress before entering the clinical in order to maintain better mental health during and after clinical practice. Secondly, our results also suggest the importance of implementing some supportive interventions to help nursing students improve their career adaptability and better adapt to the complex occupational environment. Some studies have shown that strengthening vocational education and training [37], guiding students to make correct self-evaluation and career planning [83], improving the clinical learning environment, and providing emotional and social support [40] are effective measures to help students improve their career adaptability. Finally, it is crucial to help nursing college students to establish a moderate level of professional commitment. Nursing educators and hospital administrators should come up with plans to help them maintain a stable and sustainable professional commitment, as well as provide early intervention to reduce their perceived stress and anxiety levels.

## Limitations

There were some limitations in this study. First, the convenience sampling used in this study may affect the generalizability of our results, so we should proceed with caution when interpreting our findings. Second, the sample used in this study was from a single Chinese university, and the association discovered in this study may not be representative of associations found in other universities or countries. Future research could confirm these preliminary findings in larger and more diverse populations. Third, the use of self-reporting measures may result in social desirability bias, and future studies should employ more objective measures to better reflect the participants’ true situation. Finally, the models controlled for several potential confounding variables, but other confounding factors could influence the associations. More confounding factors should be considered in future studies.

## Conclusion

Our findings further enrich the evidence that perceived stress is closely related to anxiety. Career adaptability and professional commitment influenced the relationship between perceived stress and anxiety level of nursing students. Nursing students with high career adaptability could recover from stress in clinical practice and consequently reduce their anxiety levels. Conversely, professional commitment may enhance the effect of perceived stress on anxiety. Therefore, it is critical to understand the role of career adaptability and professional commitment in promoting the mental health of nursing students and to develop targeted interventions to help nursing students to better adapt to the challenging clinical workplace.

## Data Availability

The datasets used and analyzed during the current study available from the corresponding author on reasonable request.
